# *In Vivo* Biochemical and Gene Expression Analyses of the Antioxidant Activities and Hypocholesterolaemic Properties of *Tamarindus indica* Fruit Pulp Extract

**DOI:** 10.1371/journal.pone.0070058

**Published:** 2013-07-23

**Authors:** Chor Yin Lim, Sarni Mat Junit, Mahmood Ameen Abdulla, Azlina Abdul Aziz

**Affiliations:** Department of Molecular Medicine, Faculty of Medicine, University of Malaya, Kuala Lumpur, Malaysia; Oklahoma State University, United States of America

## Abstract

**Background:**

*Tamarindus indica* (*T. indica*) is a medicinal plant with many biological activities including anti-diabetic, hypolipidaemic and anti-bacterial activities. A recent study demonstrated the hypolipidaemic effect of *T. indica* fruit pulp in hamsters. However, the biochemical and molecular mechanisms responsible for these effects have not been fully elucidated. Hence, the aims of this study were to evaluate the antioxidant activities and potential hypocholesterolaemic properties of *T. indica*, using *in vitro* and *in vivo* approaches.

**Methodology/Principal Findings:**

The *in vitro* study demonstrated that *T. indica* fruit pulp had significant amount of phenolic (244.9±10.1 mg GAE/extract) and flavonoid (93.9±2.6 mg RE/g extract) content and possessed antioxidant activities. In the *in vivo* study, hamsters fed with high-cholesterol diet for ten weeks showed elevated serum triglyceride, total cholesterol, HDL-C and LDL-C levels. Administration of *T. indica* fruit pulp to hypercholesterolaemic hamsters significantly lowered serum triglyceride, total cholesterol and LDL-C levels but had no effect on the HDL-C level. The lipid-lowering effect was accompanied with significant increase in the expression of *Apo A1*, *Abcg5* and *LDL receptor* genes and significant decrease in the expression of *HMG-CoA reductase* and *Mtp* genes. Administration of *T. indica* fruit pulp to hypercholesterolaemic hamsters also protected against oxidative damage by increasing hepatic antioxidant enzymes, antioxidant activities and preventing hepatic lipid peroxidation.

**Conclusion/Significance:**

It is postulated that tamarind fruit pulp exerts its hypocholesterolaemic effect by increasing cholesterol efflux, enhancing LDL-C uptake and clearance, suppressing triglyceride accumulation and inhibiting cholesterol biosynthesis. *T. indica* fruit pulp has potential antioxidative effects and is potentially protective against diet-induced hypercholesterolaemia.

## Introduction

Hyperlipidaemia refers to increased levels of lipids in the blood, including cholesterol and triglycerides. This increase is one of the significant risk factors involved in the development of cardiovascular disease (CVD) [1] and diabetes [2]. CVD remains an important cause of mortality and morbidity worldwide [3]. Research in the area of CVD is progressing rapidly and evidence and clinical trials for new drugs to treat CVD are continuously emerging. It is well established that increased levels of blood cholesterol especially low density lipoprotein cholesterol (LDL-C) is an important risk factor for cardiovascular complications since it favours lipid deposition in blood vessels. Epidemiological studies have clearly established that reduction of total cholesterol or LDL-C is associated with decreased risk of atherosclerosis and coronary heart disease [4], [5].

Treatment of hyperlipidaemia includes dietary changes, weight reduction, exercise and lipid-lowering drugs. Nonetheless, the use of these oral medications can lead to side effects [6]. Therefore, there continues to be a high demand for new, more effective and less toxic oral hypolipidaemic drugs. Plant products are frequently considered to be less toxic and relatively free from side effects than synthetic drugs. Hence, plants play a major role in the introduction of new therapeutic agents and have received much attention as sources of biologically active substances including antioxidative, hypoglycaemic and hypolipidaemic agents.

*Tamarindus indica*, commonly known as tamarind belongs to the family *Leguminosae*. It is native to tropical Africa, but is now grown all over the tropics. The tamarind tree is a large, frost-tender evergreen tropical tree which can grow to a height of about 30 m. *T. indica* is slow-growing but long-lived and can remain productive for 150 years or longer. The brown sticky pulp is a much-valued food ingredient in many Asian recipes due to its sweet and sour taste. *T. indica* has been used in folk medicine for treating diarrhea, stomach disorder, cold, fever, jaundice and as a skin cleanser [7]. Scientifically, the extract of *T. indica* fruit pulp has been shown to have anti-fungal and anti-inflammatory activities [8]. *T. indica* fruit pulp extract has been reported to possess hypolipidaemic effects when fed to hypercholesterolaemic hamsters [9]. However, this study did not report on the potential mechanisms of actions for the hypolipidaemic effects, particularly at the molecular levels. *In vitro* analysis carried out by our group suggest that the hypolipidaemic activity of *T. indica* fruit extract involves the regulation of expression of genes associated with lipid metabolism [10]. This study was aimed to corroborate the *in vitro* findings in order to further understand the hypolipidaemic effects of *T. Indica* and its protective action against oxidative damage.

## Materials and Methods

### Reagents and Chemicals

HPLC grade solvents, acetonitrile, ethanol and methanol were purchased from Fisher Scientific (United Kingdom). Polyphenol standards were purchased from Sigma Chemical Co. (St. Louis, USA) and had purities above 95%. Fast SYBR® Green Master Mix and High Capacity RNA-to-cDNA Master Mix was sourced from Applied Biosystems (California, United States). RNAlater™ RNA stabilization reagent, RNase-free DNase set and RNeasy mini kit were obtained from Qiagen (Hilden, Germany). Catalase, glutathione peroxidase and superoxide dismutase assay kits were purchased from Cayman Chemical Co. (Michigan, USA). 2,2-diphenyl-1-1 picrylhydrazyl (DPPH), butylated hydroxytoluene (BHT), dimethylsulphoxide (DMSO), 2,4,6-tripyridyl-s-triazine (TPTZ), Trolox and cholesterol were purchased from Sigma Chemical Co. (St. Louis, USA). Potassium persulphate and 2,2′-azino-bis(3-ethylbenzothiazoline-6-sulfonic acid) (ABTS) were sourced from Fluka Chemical (Steinheim, Germany). Sodium carbonate and Folin-Ciocalteu reagent were purchased from Merck (Darmstadt, Germany).

### Preparation of the Extract of *T. indica* fruit pulp

*T. indica* fruit pulp was collected at Universiti Putra Malaysia and identified by comparison with the Voucher Specimen (KLU 45976), deposited at the Herbarium of the Institute of Biological Sciences, University of Malaya, Kuala Lumpur. The fruit pulp was air dried, ground to powder and stored at −20°C until further analyses. Extraction of the fruit pulp was performed by mixing the dried powder with 95% ethanol at a ratio of 1∶20 (g:ml), stirred for one hour, and subsequently incubated in the dark for 48 h at room temperature [11]. The resulting extract was then filtered twice using Macherey-Nagel filter paper and the ethanol was evaporated to dryness using a rotary evaporator at 37°C. For *in vitro* analyses of polyphenolic content and antioxidant activities, the extract was dissolved in 10% DMSO and stored at −20°C.

### Polyphenolic Content Analyses

Polyphenolic content was determined using Folin-Ciocalteau assay developed by Singleton and Rossi [12]. Folin-Ciocalteau reagent (1 N) was mixed with the plant extract (1.0 mg/ml) and incubated at room temperature for 5 min before the addition of a sodium carbonate (Na_2_CO_3_) solution (60 g/l). The mixture was subsequently incubated at room temperature for 2 h. Absorbance readings were taken spectrophotometrically at 765 nm. Gallic acid was used as standard and was analyzed as above. Phenolic content was expressed as mg gallic acid equivalents (GAE)/g extract.

### Flavonoid Content Analyses

Flavonoid content of the plant extract was determined using the aluminium chloride colorimetric method described by Liu and Qiu [13]. Briefly, 100 µl of plant extract (1 mg/ml) was mixed with 10 µl of 5% sodium nitrate and 10 µl of 10% aluminium chloride. The mixture was incubated for 6 min at room temperature. One hundred microlitres sodium hydroxide (1 M) was then added to the mixture and brought to a volume of 250 µl using distilled water. Absorbance of the mixture was measured at 510 nm using a spectrophotometer. Rutin was used as standard and was analyzed as above. Flavonoid content was expressed as mg of rutin equivalents (RE)/g extract.

### Analysis of Polyphenols using High Performance Liquid Chromatography (HPLC)

The *T. indica* fruit pulp extract was subjected to acid hydrolysis before analyses of polyphenolic content using HPLC [11]. The hydrolysis process was performed by mixing 20 mg of sample with 1.6 ml of methanol and 0.4 ml of 1.2 M HCl. The mixture was then hydrolyzed at 90°C for 2 h in a tightly-sealed glass reactive vial. Subsequently, the hydrolyzed sample was centrifuged for 5 min at 5000 x g. The supernatant was collected and diluted with trifluoroacetic acid (TFA) (pH 2.6) and 200 µl was injected into the HPLC.

The HPLC system consisted of a Shimadzu dual wavelength absorbance detector (SPD-20A UV-VIS), a Rheodyne 7725i manual injector that comprised a 200 µl sample loop and column oven (CTO-10AS VP) and two pumps (LC 20AC). The mobile phase consisted of TFA (pH 2.6) as solvent A and acetonitrile as solvent B. The flow rate was 0.5 ml/min. Separation of polyphenols in the extract was achieved using the following gradient condition: 7% to 40% B for 20 min, 40% to 100% B for 6 min and 100% to 7% B for 9 min, using a reversed-phased column (NovaPak C_18_, 150×3.0 mm, inner diameter 4 µm). Absorbance of the sample was monitored at 260 nm. Data acquisition and processing were performed using the Lab Solution Chromatography Manager.

### DPPH Radical Scavenging Activity

The 2,2-diphenyl-1-1picrylhydrazyl (DPPH) radical scavenging activity of the plant extract was determined according to Cos and Rajan [14]. Various concentrations of 25 µl of the ethanol extract of *T. indica* fruit pulp was mixed with 150 µl of a 0.04 mg/ml methanolic solution of DPPH. The mixture was vortexed and then incubated in the dark at room temperature for 20 min. Absorbance of the mixture was measured at 515 nm. Trolox was used as the standard and was analysed in the same manner as the sample. DPPH radical scavenging activities of the plant extracts were expressed as mmole Trolox equivalents antioxidant capacity (TEAC) per gram of extract. Ascorbic acid, butylated hydroxytoluene (BHT) and quercetin were used as positive controls. The percentage inhibition of the DPPH radicals by the plant extract was calculated using the following equation:
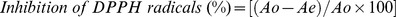
where, 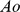 is the absorbance of the reaction mixture without the plant extract and 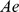 is the absorbance of the reaction mixture containing plant extract.

### ABTS Radical Scavenging Activity

The 2,2′-azino-bis(3-ethylbenzothiazoline-6-sulphonic) acid (ABTS) radical scavenging activity of the plant extract was estimated according to the method of Re and Pellegrini [15]. ABTS^+^ cation chromophore was generated by reacting 7 mM ABTS with 2.45 mM potassium persulfate for 16 h in the dark at room temperature, followed by dilution with methanol to give an absorbance of 0.70±0.02 at 734 nm. For the antioxidant assay, 10 µl of plant extract (0−125 µg/ml) was added to 1.0 ml of the ABTS reagent, incubated in the dark for 15 min before absorbance was read at a wavelength of 734 nm. Trolox was used as the standard and analysed in the same manner as the sample. ABTS radical-scavenging activities of the plant extracts were expressed as mmole Trolox equivalents antioxidant capacity (TEAC) per gram of extract. Ascorbic acid, BHT and quercetin were used as positive controls. The percentage inhibition of the ABTS radicals by the plant extract was calculated using similar equation as the DPPH radical scavenging activity.

### Ferric Reducing Antioxidant Power (FRAP) Activity

The ferric reducing activity of the plant extract was estimated using the method developed by Benzie and Strain [16]. Reagents for the assay consisted of 300 mmol/l acetate buffer, 10 mmol/l 2,4,6-tripyridyl-s-triazine (TPTZ) in 40 mmol/l of HCl and 20 mmol/l of FeCl_3_.6 H_2_O. The working FRAP reagent was prepared fresh by mixing 25 ml of acetate buffer, 2.5 ml of TPTZ solution and 2.5 ml of FeCl_3_.6 H_2_O. The freshly prepared mixture was incubated at 37°C in a water bath for 5 min before a blank reading was taken spectrophotometrically at 593 nm. After that, 30 µl of plant extract or standard and 90 µl of distilled water were added to 900 µl of the FRAP reagent. Absorbance of the mixture was measured immediately and thereafter at an interval of 15 sec for 4 min. The change in absorbance in the 4 min time reaction was calculated. A calibration curve was generated using ferrous sulphate as the standard. Ascorbic acid, BHT and quercetin were used as positive controls and analysed as above. Ferric reducing activity was expressed as µmole Fe(II) per gram of extract.

### Ethics Statement for Animal Study

The protocol for the *in vivo* study was conducted with the approval of the ethics committee for animal experiments, Faculty of Medicine, University of Malaya {ethics no: PM/26/08/2009/AAZ(R)}.

### Animal Diet

*T. indica* fruit pulp extract (500 mg/kg body weight) for the feeding study was prepared by diluting the dried fruit pulp powder with distilled water. High-cholesterol diet (1%) was prepared by mixing the standard chow with cholesterol. Briefly, standard chow (99 g) was ground into powder and mixed with 1 g of cholesterol, after which 100 ml of distilled water was added and the mixture was shaped into small pellets. The pellets were dried in an oven at 50°C and kept at 4°C before use.

### Treatment of Hamsters with *T. indica* Fruit pulp Extract

Six week-old male Syrian hamsters (n = 20) were randomly divided into four treatment groups and housed individually in well-ventilated cages under a 12-h light:dark cycle. Diet and water were provided *ad libitum*. Animals were treated for ten weeks with the following diet; Group I: standard chow plus distilled water (5 ml/kg body weight); Group II: standard chow plus *T. indica* fruit pulp extract (500 mg/kg body weight); Group III: high-cholesterol diet plus distilled water (5 ml/kg body weight) and Group IV: high-cholesterol diet plus *T. indica* fruit pulp extract (500 mg/kg body weight). Feeding of *T. indica* fruit pulp extract was conducted through the gavage method once a day.

### Collection and Preparation of Blood and Liver Samples

At the end of the feeding experiment, the animals were anaesthetized with a ketamine-xylazine cocktail (90 mg/kg and 2 mg/kg body weight, respectively) and blood was collected through cardiac puncture after an overnight fast. The livers of the animals were excised, homogenized with cold phosphate buffered saline, centrifuged at 2000 x g at 4°C for 15 min and the supernatant stored at −80°C before analysis. For the gene expression study, 1 g of the liver tissues were immediately washed with saline, kept in RNAlater™ RNA Stabilization Reagent (Qiagen) and stored at −80°C until further analysis.

### Biochemical Analysis

Serum samples were analysed for lipid profiles, fasting blood glucose, alanine aminotransferase (ALT) and aspartate aminotransferase (AST) levels using commercially available kits on an automated analyser.

### Analyses of Antioxidant Enzyme Activities

Activities of the antioxidant enzymes, catalase (CAT), glutatione peroxidase (GPx) and superoxide dismutase (SOD) were determined using commercially available assay kits (Cayman, USA). The assays were performed according to the manufacturer’s instructions.

### Determination of Antioxidant Activities and TBARS in Serum and Liver

The ABTS radical scavenging and FRAP activities of serum and liver homogenate were analysed as described above. Thiobarbituric acid reactive substance (TBARS) assay was used to assess lipid peroxidation in serum and hepatic samples by measuring levels of malondialdehyde (MDA) [17]. A mixture of 100 µl sample and 100 µl trichloroacetic acid (10%) was centrifuged at 3500 rpm at 25°C for 5 min. One hundred and twenty microlitres of 0.67% thiobarbituric acid was added to the clear supernatant and heated at 100°C for 15 min and then cooled at room temperature. Absorbance of the mixture was read at 550 nm. 1,1,3,3-tetraethoxypropane was used as standard. Lipid peroxidation was expressed as nmol MDA/ml.

### Gene Expression Analysis

The expression of selected genes that were associated with antioxidant activity and lipid metabolism (Table 1) were quantitated using real time relative quantitative polymerase chain reaction (qRT-CR) as previously described [10].

**Table 1:** Primer sequences used for qRT-PCR analysis of selected genes in hamsters.

Gene (GenBank accession no.)	Forward primer (5′–3′)	Reverse primer (5′–3′)	Size of PCR product
*Apo A1* (AF046919.1)	CGCCACCACGTTGACGCTCT	GGTCAGCGGCCTTGGTGTGG	133
*Beta actin* (AJ312092.1)	CGACAACGGCTCCGGCATGT	TCACGCCCTGGTGCCTAGGG	101
*Abcg5* (NM_053754.2)	GGAAGGGGAGGTGTTTGT	GCCAGCATCGCCGTGTAG	138
Abcg8 (NM_130414.2)	CATCATTGGCTTCCTTTA	CCGCTCCGAGTGACATTT	139
*Cyp1A1* (D12977.1)	TAAAGCACGCCCGCTGCGAA	AGC CCCCTGCTCTGGTGACC	103
*Gstm1* (M59772.1)	AGCTGGGCCTGGACTTCCCC	ACACAGGTCGTGCTTGCGGG	107
*HMG-CoA Reductase* (M12705.1)	GCTGTCTGGTGGCCAGCACC	GGAAGACGCACCACTGGGCC	112
*LDL-C Receptor* (M94387.1)	GGCAGCGCTGACTGCAAGGA	TTCACGGTCACACTGGCGGC	123
*Mtp* (U14995.1)	AGCTGGCCTGGAGAGCAGGG	GCCCGGTCCATCTGCATGCA	105

*Apo A1*, Apolipoprotein A1; *Abcg5*, ATP-binding cassette, subfamily G (WHITE), member 5; *Abcg8*, ATP-binding cassette, subfamily G (WHITE), member 8; *Cyp1A1*, Cytochrome P450, family 1, subfamily A, polypeptide 1; *Gstm1*, Glutathione S-transferase mu 1; *Mtp*, Microsomal triglyceride transfer protein.

### RNA Extraction, Cleanup and Quantitation

Total cellular RNA (tcRNA) was extracted from 30 mg of the liver of control and treated hamsters using the RNeasy Mini Kit and its accompanying QIAshredder (Qiagen) in accordance with the manufacturer’s instructions. Briefly, the tissues were thoroughly ground to fine powder in liquid nitrogen with a mortar and pestle. Highly denaturing guanidine thiocyanate-containing buffer, buffer RLT was then added to the powder and the cell suspension was homogenized using QIAshredder. The homogenate was centrifuged and the lysate was then transferred to a microcentrifuge tube. The lysate was centrifuged and the supernatant was collected. Ethanol (50%) was then added to the supernatant and mixed well by pipetting. The mixture was then transferred to an RNeasy Spin Column and washed with RW1 and RPE buffers (QIAGEN; composition not declared). RNase-free water (40 µl) was added to the RNeasy Spin column and the eluent containing the tcRNA was treated with DNase I for 10 min to remove contaminating DNA using RNase-Free DNase Set (Qiagen). Then, buffer RLT and absolute ethanol were added to the samples and the mixture was transferred to an RNeasy Spin Column. Buffer RPE was added to wash the spin column membrane. In the final step, tcRNA was eluted with RNase-free water into collection tubes. Absorbance of the extracted tcRNA was measured at 260 and 280 nm using GeneQuantpro spectrophotometer. The concentration of the tcRNA was quantified using the 260 nm absorbance reading while its purity was evaluated from the 260∶280 ratio.

### Reverse Transcription of the tcRNA

TcRNA (1000 ng) was reverse-transcribed into complementary DNA (cDNA) using a High Capacity RNA-to-cDNA Master Mix (Applied Biosystems) following the manufacturer’s instructions. The final reaction volume for the reverse transcription was 20 µl, consisted of 1000 ng TcRNA and 1 X Master Mix which contained magnesium chloride (MgCl_2_), deoxyribonucleotide triphosphate (dNTPs), recombinant RNase inhibitor protein, reverse transcriptase, random primers, oligo(dT) primer and stabilizers. The mixture was pipetted into a microcentrifuge tube and then placed in a thermocycler with the following program: 25°C for 5 min, 42°C for 30 min and 85°C for 5 min. The cDNA was kept at −80°C until further analysis.

### Real-time Relative Quantitative RT–PCR (qRT–PCR)

Oligonucleotide primers used for qRT-PCR are listed in Table 1. All cDNA samples were diluted to a final concentration of 5 ng/µl. The final reaction volume for real time PCR was 20 µl, consisted of 10 µl of 2 X Fast SYBR® Green Master Mix (Applied Biosystem) which contained SYBR® Green I Dye, AmpliTaq® Fast DNA Polymerase, UP (Ultra Pure), Uracil-DNA Glycosylase (UDG), ROX™ dye Passive Reference, dNTPs and optimized buffer components, 200 nM of forward primer, 200 nM of reverse primer and 10 ng of cDNA. qRT-PCR was performed using Applied Biosystems StepOne Plus™ Real Time PCR System with a thermal profile consisted of 20 sec of *Taq* DNA Polymerase activation at 95°C, followed by 40 cycles of denaturation at 95°C for 3 sec, primer annealing at 60°C for 30 sec. The melting curves of the real-time PCR products were analysed from 65°C to 95°C to aid in product identification. Each measurement was carried out in triplicate. Differences in gene expression, expressed as fold-change, were calculated using the 2^–ΔΔCt^ method where *beta actin* was used as the reference gene.

### Statistical Analyses

All analyses were done in triplicate and data were expressed as means ± standard error of means. Significant differences among the groups were determined by one-way ANOVA using SPSS followed by the post hoc Tukey’s Honestly Significant Different test. Values corresponding to *p*<0.01 or *p*<0.05 were considered to be statistically significant.

## Results

### Polyphenolic and Flavonoid Content and Antioxidant Activities of *T. indica* Fruit pulp

The *T. indica* fruit pulp extract contains considerable amounts of polyphenols and flavonoids (Table 2) and showed moderate antioxidant activities, which were comparable to the positive control BHT but lower than ascorbic acid and quercetin.

**Table 2:** Polyphenol, flavonoid and antioxidant activities of the ethanol extract of *T. indica* fruit pulp.

	Polyphenolic content	Flavonoid content	DPPH	ABTS	FRAP
*T. indica* fruit pulp	244.93±10.07	93.93±2.57	0.08±0.01^c^	0.07±0.04^b^	0.72±0.03^b^
Ascorbic acid	NA	NA	4.05±0.02^a^	1.13±0.04^a^	6.52±0.10^a^
BHT	NA	NA	0.09±0.02^c^	0.14±0.04^b^	0.70±0.07^b^
Quercetin	NA	NA	3.94±0.02^b^	2.93±0.06^a^	6.46±0.08^a^

Values are expressed as mean ± standard deviation (n = 3). Means with different superscript letters within the same column are significantly different (*p*<0.05). Polyphenolic content is expressed as mg GAE/g extract; flavonoid content is expressed as mg RE/g extract; DPPH, 1,1-diphenyl-2-picryl hydrazyl radical scavenging activity and ABTS, 2,2′-azino-bis(3-ethylbenzothiazoline-6-sulphonic acid) radical scavenging activity are expressed as mmole TE/g extract; FRAP, ferric reducing antioxidant power is expressed as mmole of Fe(II)/g extract; NA, not available.

**Figure 1:**
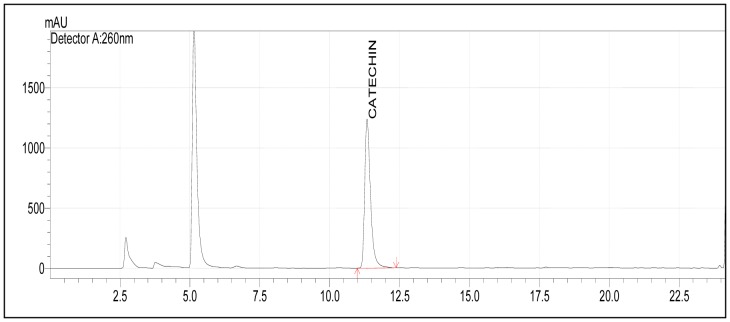
Chromatogram of *T. indica* fruit pulp analysed by HPLC. HPLC analysis was carried out on the hydrolysed sample of *T. indica* fruit pulp using a reversed-phase column (NovaPak C_18_, 150 x 3.0 mm, inner diameter 4 µm). Separation of polyphenols was achieved using a linear gradient system comprising of acetonitrile in trifluoroacetic acid (pH2.6) as the mobile phase. Absorbance was measured at 260 nm.

Figure 1 shows the chromatogram of hydrolysed *T. indica* fruit pulp analysed by HPLC. Catechin was identified in the fruit pulp sample by comparing the retention times of the peak with the authentic standard.

### Body Weight and Liver Weight of Control and Treated Hamsters

Table 3 shows the food consumption, body weight and weight of livers of the hamsters. No significant differences were seen in the amount of food consumption among the four groups. With the exception of the hypercholesterolaemic group (Gp. III), the remaining groups showed reduced weight after the 10-week experimental period. There were no significant differences in weight of livers among the control hamsters, hamsters treated with *T. indica* fruit pulp and hypercholesterolaemic hamsters treated with *T. indica* fruit pulp. On the contrary, the hypercholesterolaemic hamsters showed significant increase in liver weight, with the weight twice those of the control group (*p*<0.01). However, administration of *T. indica* fruit pulp to hypercholesterolaemic hamsters reduced liver weight by approximately 35% relative to the hypercholesterolaemic hamsters.

**Table 3:** Food consumption, body weight and liver weight of control and hamsters treated with *T. indica* fruit pulp extracts, in the presence or absence of cholesterol.

Group	Group I	Group II	Group III	Group IV
Food Consumption (g/day)	8.71±0.32	8.72±0.21	8.51±0.40	8.22±0.24
Intial body weight(g)	128.80±5.70	137.00±5.15	127.80±1.39	135.40±2.23
Final body weight (g)	121.60±5.26	130.80±3.99	139.00±4.14	127.00±1.34
Weight change (g)	- 7.20±1.69^b^	- 6.20±4.60^b^	+7.00±0.71^a^	- 5.20±0.45^b^
Liver weight (g)	3.40±0.24^b^	3.60±0.24^b^	6.80±0.20^a^	4.40±0.24^b^

Values are given as mean ± standard error of mean (n = 5). Values not sharing a common superscript letter within the same row differ significantly at *p*<0.01. Group I: standard chow plus distilled water (5 ml/kg body weight); Group II: standard chow plus *T. indica* fruit pulp extract (500 mg/kg body weight); Group III: high-cholesterol diet plus distilled water (5 ml/kg body weight) and Group IV: high-cholesterol diet plus *T. indica* fruit pulp extract (500 mg/kg body weight).

### Effect of *T. indica* Fruit pulp on Biochemical Parameters in Hamsters

Table 4 summarizes the serum lipid profiles, glucose, AST and ALT levels of control and hamsters treated with *T. indica* fruit pulp. There was no significant difference in levels of serum triglyceride, total cholesterol, HDL, LDL, glucose, AST and ALT between control hamsters (Gp. I) and hamsters treated with *T. indica* fruit pulp (Gp. II). Feeding a high-cholesterol diet to the hamsters significantly increased all the biochemical parameters. On the contrary, when *T. indica* fruit pulp was fed together with the high-cholesterol diet, levels of triglyceride and LDL-C reduced to amounts similar to the normal untreated group whereas the level of total cholesterol reduced to roughly 50% compared to the control group. However, there was no significant change in HDL levels between the treated and untreated hypercholesterolaemic groups.

**Table 4:** Analyses of biochemical parameters of control and hamsters treated with *T. indica* fruit pulp extracts, in the presence or absence of cholesterol.

Group	Group I	Group II	Group III	Group IV
Triglyceride (mmol/l)	0.88±0.05^b^	1.00±0.10^b^	1.98±0.21^a^	0.96±0.09^b^
Total choleste-rol (mmol/l)	2.08±0.12^c^	1.94±0.08^c^	5.74±0.55^a^	3.74±0.14^b^
HDL-C (mmol/l)	1.34±0.07^b^	1.25±0.07^b^	2.20±0.23^a^	2.27±0.19^a^
LDL-C (mmol/l)	0.34±0.08^b^	0.21±0.07^b^	2.38±0.56^a(*)^	0.95±0.15^b(*)^
Glucose (mmol/l)	8.38±0.62^ab^	5.18±0.27^b^	10.00±2.00^a^	8.66±0.78^ab^
AST (IU/l)	95.20±13.38^a^	70.20±12.73^a^	142.00±29.62^a^	87.00±9.47^a^
ALT (IU/l)	51.40±5.81^b^	62.80±11.21^b^	243.00±84.10^a^	104.60±22.69^ab^

Values are given as mean ± standard error of mean (n = 5). Values not sharing a common superscript letter within the same row differ significantly at *p*<0.01 and ^*^ *p*<0.05. Group I: standard chow plus distilled water (5 ml/kg body weight); Group II: standard chow plus *T. indica* fruit pulp extract (500 mg/kg body weight); Group III: high-cholesterol diet plus distilled water (5 ml/kg body weight) and Group IV: high-cholesterol diet plus *T. indica* fruit pulp extract (500 mg/kg body weight).

The levels of glucose only increased in hypercholesterolaemic hamsters (Gp. III) and remained unchanged in the other groups. Although hamsters treated with *T. indica* fruit pulp showed reduced glucose levels compared to control group, the values were not significantly different. There was no significant difference in AST activities among the four groups. Hypercholesterolaemic hamsters showed significant increase in serum ALT compared to control hamsters. Administration of *T. indica* fruit pulp to the hypercholesterolaemic hamsters appeared to reduce ALT levels relative to the hypercholesterolaemic hamsters, however this was not statistically significant.

### Effects of *T. indica* Fruit pulp on Serum and Liver Oxidative Status in Hamsters

Serum and liver antioxidant enzymes, antioxidant activities and lipid peroxidation levels are presented in Table 5 and 6, respectively. Hamsters fed the high-cholesterol diet showed reduced activities of CAT, SOD and GPx in serum and liver. Interestingly, treatment of either the control or hypercholesterolaemic hamsters with *T. indica* fruit pulp significantly increased activities of CAT and SOD in both serum and liver. In contrast, activity of GPx was unchanged in the serum of control hamsters fed *T. indica* fruit pulp although there was increased GPx activities in the hypercholesterolaemic hamsters fed *T. indica* fruit pulp in both serum and liver and also in the liver of control group fed *T. indica* fruit pulp.

**Table 5:** Serum antioxidant enzymes, antioxidant activities and lipid peroxidation in control and hamsters treated with *T. indica* fruit pulp extracts, in the presence or absence of cholesterol.

Group	Group I	Group II	Group III	Group IV
Catalase (µmol/min/ml)	3.54±0.21^b^	4.48±0.19^a^	2.67±0.04^c^	3.24±0.04^b^
Superoxide dismutase (U/ml)	13.34±0.63^b^	18.67±0.47^a^	6.25±0.67^c^	12.00±0.45^b^
Glutathione peroxidase(µmol/min/ml)	0.60±0.02^a^	0.63±0.05^a^	0.22±0.01^c^	0.43±0.02^b^
ABTS (µmol/ml serum)	2.53±0.08^b^	2.93±0.03^a^	2.14±0.05^c^	2.41±0.05^b^
FRAP (µmol Fe (II)/ml serum)	0.32±0.02^b^	0.40±0.01^a^	0.28±0.01^b^	0.30±0.01^b^
MDA (nmol/ml serum)	0.033±0.001	0.033±0.001	0.036±0.002	0.034±0.001

Values are given as means ± standard error of mean (n = 5). Values not sharing a common superscript letter within the same row are significantly different at *p*<0.05. Group I: standard chow plus distilled water (5 ml/kg body weight); Group II: standard chow plus *T. indica* fruit pulp extract (500 mg/kg body weight); Group III: high-cholesterol diet plus distilled water (5 ml/kg body weight) and Group IV: high-cholesterol diet plus *T. indica* fruit pulp extract (500 mg/kg body weight). DPPH, 1,1-diphenyl-2-picryl hydrazyl radical scavenging activity; ABTS, 2,2′-azino-bis(3-ethylbenzothiazoline-6-sulphonic acid) radical scavenging activity; FRAP, ferric reducing antioxidant power; MDA (malondialdehyde).

**Table 6:** *Hepatic* antioxidant enzymes, antioxidant activities and lipid peroxidation in control and hamsters treated with T. indica fruit pulp extracts, in the presence or absence of cholesterol.

Group	Group I	Group II	Group III	Group IV
Catalase (µmol/min/ml)	1279.25±34.48^b^	1857.16±49.07^a^	958.65±44.48^c^	1197.41±19.54^b^
Superoxide dismutase(U/ml)	3616.48±67.98^b^	4452.17±139.81^a^	3153.66±110.03^c^	3592.95±87.34^b^
Glutathione peroxidase (µmol/min/ml)	8.48±0.63^b^	10.70±0.33^a^	6.91±0.20^c^	8.70±0.43^b^
ABTS (µmol/g liver)	16.93±0.52^b^	19.67±0.38^a^	11.35±0.56^d^	13.32±0.27^c^
FRAP (µmol Fe (II)/g liver)	3.26±0.07^a^	3.43±0.08^a^	2.07±0.10^c^	2.48±0.09^b^
MDA (nmol/g liver)	0.48±0.04^c^	0.46±0.03^c^	1.82±0.06^a^	1.18±0.07^b^

Values are given as means ± standard error of mean (n = 5). Values not sharing a common superscript letter within the same row differ significantly at *p*<0.05. Group I: standard chow plus distilled water (5 ml/kg body weight); Group II: standard chow plus *T. indica* fruit pulp extract (500 mg/kg body weight); Group III: high-cholesterol diet plus distilled water (5 ml/kg body weight) and Group IV: high-cholesterol diet plus *T. indica* fruit pulp extract (500 mg/kg body weight). DPPH, 1,1-diphenyl-2-picryl hydrazyl radical scavenging activity; ABTS, 2,2′-azino-bis(3-ethylbenzothiazoline-6-sulphonic acid) radical scavenging activity; FRAP, ferric reducing antioxidant power; MDA (malondialdehyde).

The serum and liver of hypercholesterolaemic hamsters showed reduced ABTS radical scavenging activities although treatment of either the control or hypercholesterolaemic group with *T. indica* fruit pulp significantly increased ABTS radical scavenging activities in both serum and liver. In serum, FRAP values did not differ among the control, hypercholesterolaemic and hypercholesterolaemic group treated with *T. indica* fruit pulp. However, hamsters treated with *T. indica* fruit pulp (Gp. II) showed 20% higher FRAP activities compared to control (Gp. I). In the liver, administration of *T. indica* fruit pulp did not cause any significant change in FRAP activities compared to control group. However, in the hypercholesterolaemic hamsters fed *T. indica* fruit pulp, FRAP activities increased by approximately 20% compared to hypercholesterolaemic hamsters (*p*<0.05).

There was no significant difference in serum MDA levels among all the groups. Similarly, lipid peroxidation in the liver of the control (Gp. I) and *T. indica* fruit pulp-treated (Gp. II) hamsters was not significantly altered. High-cholesterol diet induced lipid peroxidation in the liver while treatment of *T. indica* fruit pulp to hypercholesterolaemic hamsters lowered lipid peroxidation by approximately 35%.

### Effects of Ethanolic Extract of *T. indica* Fruit pulp on the Expression of Selected Hepatic Genes Associated with Lipid Metabolism and Antioxidant Activity in Hamsters

**Figure 2:**
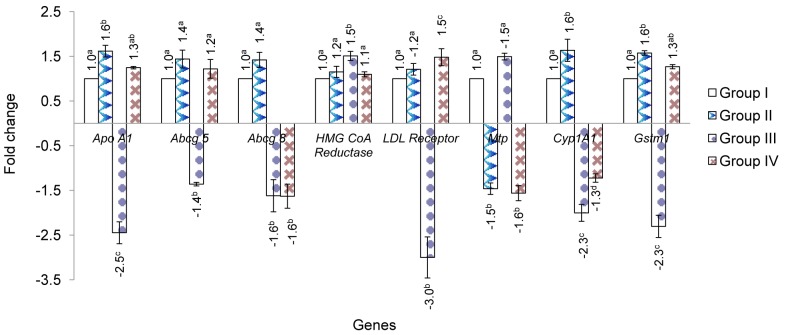
The effect of ethanolic extract of *T. indica* fruit pulp on the expression of selected hepatic genes in hamster (n = 3). Each bar represents mean ± standard error of mean. Values not sharing a common superscript letter differ significantly at *p*<0.05. Group I: standard chow plus distilled water (5 ml/kg); Group II: standard chow plus 500 mg/kg *T. indica* fruit pulp extract; Group III: high-cholesterol diet plus distilled water (5 ml/kg). Group IV: high-cholesterol diet plus 500 mg/kg *T. indica* fruit pulp extract.

In hypercholesterolaemic hamsters, the expression of the *Abcg5* was significantly down-regulated by 1.4-fold and the suppression of the *ApoA-I* gene expression was also significantly reversed when the hypercholesterolaemic hamsters were co-treated with *T. indica* extract. Significant down-regulation was observed for *Abcg8* gene expression in the hypercholesterolaemic hamsters. Unlike the *Abcg5,* the expression of *Abcg8* in the hypercholesterolaemic hamsters was unchanged in the presence of *T. indica* extract. Both *Abcg5* and *Abcg8* were up-regulated by 1.4-fold in hamsters treated with only *T. indica* fruit pulp extract (Gp. II). Besides that, *HMG-CoA reductase* gene expression was significantly up-regulated by 1.5-fold with the hypercholesterolaemic diet relative to that of control. *T. indica* extract managed to significantly suppress the *HMGCoA reductase* gene expression in the presence of cholesterol. The expression of *HMG-CoA reductase* was not significantly altered in hamsters receiving only *T. indica* fruit pulp extract.

*LDL receptor* gene was significantly down-regulated by 3.0-fold (*p*<0.05) in the presence of high cholesterol diet. When given together with *T. indica* fruit extract, the regulation of gene expression was reversed. However, treatment with only *T. indica* fruit extract did not significantly regulate the expression of *LDL receptor* gene.

The expression of microsomal triglyceride transfer protein (*Mtp*) was down-regulated by 1.5-fold in *T. indica*-treated hamsters (Gp. II) compared with control hamsters (Gp. I). Meanwhile, hamsters fed the high-cholesterol diet had increased hepatic expression level of *Mtp* gene by 1.5-fold compared with control hamsters, whereas, *T. indica* fruit pulp extract administration to hypercholesterolaemic hamsters caused down-regulation of *Mtp* gene expression level by 1.6-fold relative to the control hamsters.

In *T. indica* fruit pulp extract supplemented hamsters, the expression level of *Cyp1A1* gene was significantly (*p*<0.05) increased by 1.6-fold relative to control hamsters. In contrast, hepatic *Cyp1A1* expression was down-regulated by 2.3-fold in hamsters fed the high-cholesterol diet. Interestingly, treatment of *T. indica* fruit pulp extract to hypercholesterolaemic hamsters for ten weeks significantly (*p*<0.05) increased *Cyp1A1* level compared to hypercholesterolaemic hamsters. In addition, the gene expression of *Gstm1* was significantly up-regulated by 1.6-fold with the feeding of *T. indica* fruit pulp extract to control hamsters. In hypercholesterolaemic hamsters, the hepatic gene expression of *Gstm1* was significantly (*p*<0.05) reduced (2.3-fold) compared with control. Surprisingly, the administration of *T. indica* fruit pulp extract along with high-cholesterol diet up-regulated *Gstm1* gene expression level by 1.3-fold relative to control hamsters.

## Discussion

A few studies have reported various biological activities of *T. indica* [7], [8]. Our group recently reported the high antioxidant activities of various parts of *T. indica* [11] as well as its ability to influence expression of genes and proteins which may explain its reported biological activities [10], [18]. Although the antioxidant activity of the fruit pulp in this study was lower than that of the leaves [13], the fruit pulp was chosen for the animal study as it was the part of the plant that was edible and commonly consumed. *T. indica* fruit pulp contains considerable amounts of phenolics and flavonoids as well as antioxidant activities. The antioxidant activities demonstrated by the fruit pulp could be due to the presence of phenolic compounds. Catechin, which was detected in the fruit pulp by HPLC, had been reported to have strong antioxidant activity [19]. In addition to catechin, Sudjaroen and Haubner [20] identified other phenolics in *T. indica* fruit pulp, including epicatechin, taxifolin, apigenin, eriodictyol, luteolin, naringenin and different classes of procyanidins. These compounds were reported to exhibit strong antioxidant properties and lipid-lowering effects [21], [22].

Epidemiological studies have shown inverse relationships between the intake of flavonoids from vegetables and fruits and the incidence of heart disease [23], [24]. Hence, many plants have been investigated for their potential hypocholesterolaemic properties. Hamsters are a suitable animal model for studying cholesterol metabolism due to their ability to develop hypercholesterolaemia when fed a high-cholesterol diet [9] in addition to having similar characteristics of cholesterol metabolism as humans [25].

The increase in liver weight of cholesterol-fed hamsters could be due to accumulation of cholesterol and triglyceride as a result of increased absorption from the diet. Liver is the major site for cholesterol metabolism in hamsters as well as humans [26] and a high-cholesterol diet had been reported to increase liver weight of hamsters [27], [28]. However, feeding of *T. indica* fruit pulp along with the high-cholesterol diet reversed this condition, possibly as a result of increased excretion and/or metabolism of cholesterol. The increased body weight of hypercholesterolaemic hamsters was likely contributed by the increase in liver weight. Serum AST, indicators of hepatotoxicity, were unchanged in hamsters treated with *T. indica* fruit pulp. Although ALT increased in hypercholesterolaemic hamsters, treatment with *T. indica* fruit pulp reduced the levels back to normal. Increased ALT in hamsters fed a high-cholesterol diet is commonly observed [29], [30]. Martinello and Soares [9] reported that administration of *T. indica* fruit pulp to hamsters did not lead to changes in AST and ALT levels, indicating that *T. indica* fruit pulp is not hepatotoxic.

In this study, hamsters fed on the high-cholesterol diet (Gp. III) showed increased triglyceride, total cholesterol, HDL-C and LDL-C compared to normal hamsters (Gp. I). A high-cholesterol diet can reduce the LDL-C clearance rate of hamsters causing increased LDL-C in the circulation [26]. Decreasing cholesterol and triglyceride levels in blood prevent the risk of cardiovascular diseases and atherosclerosis [31]. In this study, administration of *T. indica* fruit pulp together with high-cholesterol diet decreased triglyceride, total cholesterol and LDL-C but did not alter HDL-C levels. It is interesting to note that feeding *T. indica* fruit pulp to hypercholesterolaemic hamsters reduced LDL-C by more than two-fold. This suggests that the fruit pulp has cholesterol-lowering effect but it does not involve HDL-C directly.

Taxifolin, apigenin, naringenin and luteolin, phenolic compounds identified in *T. indica* fruit pulp had been reported to have hypolipidaemic properties, including inhibiting the production of cholesterol, suggesting their possible contribution towards improving the serum lipid profiles in cholesterol-fed hamsters [21], [22], [32].

Serum HDL-C levels were significantly higher in the hypercholesterolaemic hamsters (Gp. III) compared to those on the normal diet (Gp. I). This observation is in agreement with previous studies in humans and hamsters where high-cholesterol diet caused HDL-C elevation [33], [34]. In addition, the increased HDL-C levels in cholesterol-fed hamsters could also be an adaptive mechanism showing the need to enhance cholesterol efflux through the reverse cholesterol transport pathway in response to the high-cholesterol diet. Moreover, administration of *T. indica* fruit pulp along with the high-cholesterol diet did not produce significant variation in the serum HDL-C levels compared to hamsters fed on the high-cholesterol diet which suggests that *T. indica* fruit pulp had no effect on HDL-C.

A few genes that may contribute to the hypocholesterolaemic effect of *T. indica* fruit pulp were chosen and their expressions were determined by qRT-PCR to evaluate the molecular mechanisms underlying its lipid-lowering effects. We had previously shown that the *T. indica* fruit pulp extracts were able to regulate the expression of genes associated with lipid metabolism in an *in vitro* model, liver HepG2 cells [10]. Amongst the regulated genes were those encoding ABCG5, ApoA-1 and MTP. In the same study, the *T. indica* fruit pulp extract did not alter the expression of genes encoding LDL receptor which facilitates cholesterol uptake from the blood circulation as well as HMG-CoA reductase, the rate-limiting enzyme in the *de novo* synthesis of cholesterol. In this study, the *HMG-CoA reductase* gene expression in hypercholesterolaemic hamsters increased in parallel with serum total cholesterol concentration. This result is consistent with previous reports where animals fed a high-cholesterol diet had elevated *HMG-CoA reductase* expression [35], [36]. This finding points out the potential role of exogenous cholesterol as a cause for the up-regulation of hepatic *HMG-CoA reductase* expression. The observed increase in gene expression could also be due to the elevated glucose concentration in hamsters fed the high-cholesterol diet since insulin function to increase hepatic *HMG-CoA reductase* activity [37], [38].

Consistent with our *in vitro* analysis, hamsters treated with only *T. indica* fruit pulp extract did not show significant changes in the expression of *HMG-CoA reductase* gene compared to the control group. However, hamsters fed on the high-cholesterol diet along with *T. indica* fruit pulp had decreased expression level of *HMG-CoA reductase* compared to hypercholesterolaemic hamsters. This group of animals also had decreased levels of serum triglyceride, total cholesterol and LDL-C. This indicates that in the presence of cholesterol, the hypolipidaemic effect of *T. indica* fruit pulp could have possibly reduced cholesterol levels by reducing hepatic *HMG-CoA reductase* gene expression. Taxifolin had also been shown to have lipid-lowering effects through inhibition of the HMG-CoA reductase activity in HepG2 cells [21]. Therefore, the lipid lowering effect of *T. indica* fruit pulp could be due to the presence of taxifolin through the inhibition of *HMG-CoA reductase* expression.

The metabolism of LDL-C is greatly influenced by the number and activity of LDL receptors [39], [40]. Hence, functional LDL receptors are vital to take up LDL-C in order to decrease their circulating levels in the blood circulation which is one of the contributing factors towards the development of atherosclerosis. The effect of the plant extract on the expression of *LDL receptor* gene was studied to ascertain if the cholesterol-lowering effect of the plant extract occurred through increased production of LDL receptors. In the present study, the gene expression of *LDL receptor* in *T. indica* fruit pulp-fed hamsters was not significantly different from control hamsters. Similarly, the serum concentration of LDL-C in both groups of animals was not significantly different. The expression of *LDL receptor* in hamsters fed on the high-cholesterol diet was significantly down-regulated, signifying the possibility that high intracellular cholesterol reduced and suppressed synthesis of LDL receptors. LDL-C in excess was not efficiently taken up by the limited amounts of LDL receptors, hence decreasing LDL-C clearance from peripheral tissues [35], [41]. This could explain the elevation of serum LDL-C observed in the present study. This finding is consistent with another study in hamsters [42] where high-cholesterol diet caused down-regulation of *LDL receptor* expression. On the other hand, administration of *T. indica* fruit pulp to hypercholesterolaemic hamsters significantly up-regulated the hepatic mRNA levels of *LDL receptor*. This result suggests that *T. indica* fruit pulp could have induced *LDL receptor* gene expression, causing increased uptake and clearance of LDL-C from peripheral tissues, thus decreasing serum LDL-C concentration in hypercholesterolaemic hamsters. Indeed, plant extracts have been reported to be able to lower LDL-C levels by increasing LDL receptor activities [43]. It was reported that HepG2 cells treated with cacao polyphenols such as catechin elevated LDL receptor activity [44]. Catechin which is also present in the *T. indica* fruit pulp could have contributed towards reducing LDL-C in the hypercholesterolaemic hamsters by inducing the expression of *LDL receptor.*

Another gene that was measured in this study was *Apo A1* which codes for Apolipoprotein A1 (Apo A1). Apo A1 is one of the major lipoprotein constituents of HDL-C and is involved in cholesterol efflux from tissues to liver for excretion [45]. Some studies have reported inverse relationship between serum Apo A1 levels and CHD [46], [47]. Similarly, over-expression of *Apo A1* in *Apo A1* transgenic mice has been shown to increase HDL-C and reduce the risk against diet-induced atherosclerosis [48]. The possible mechanism exerted by Apo A1 to provide cardioprotective effects include its role in reversing cholesterol transport which is the main pathway to eliminate excess cholesterol from peripheral tissues and to transport them to the liver. Cholesterol in the liver is then partially converted into bile acids and subsequently excreted in bile. Thus, the concentration of HDL-C is highly correlated with Apo A1 levels and *Apo A1* gene expression may be an essential determinant of HDL-C. Therefore, increasing the production of Apo A1 by enhancing its gene transcription in the liver would be greatly advantageous.

In this study, hepatic expression of *Apo A1* was down-regulated in hamsters fed with high-cholesterol diet compared with control, indicating that the cholesterol-enriched diet suppressed the *Apo A1* expression which could have led to the observed elevated total cholesterol and triglyceride levels. A report showed that high-cholesterol diet decreased the levels of Apo A1 in rabbits [49]. However, in this study, the down-regulation of *Apo A1* expression was not accompanied by a decreased in HDL-C levels in the hypercholesterolaemic hamsters. This is unexpected because suppression of *Apo A1* gene is predicted to also decrease HDL-C levels. It is possible that other mechanisms maybe involved in regulating HDL-C levels. For instance, overexpression of lecithin cholesterol acyltransferase (LCAT) in hamsters had been reported to increase HDL-C levels [50]. *T. indica* fruit pulp supplementation to normal hamsters neither altered the expression of hepatic *Apo A1* gene nor the levels of HDL-C. However, in hypercholesterolaemic hamsters, *T. indica* fruit pulp was able to up-regulate *Apo A1* gene expression as well as increase HDL-C levels suggesting the role of the extract in reverse cholesterol transport mechanism.

Reverse cholesterol transport also includes the secretion of excess cholesterol in the liver into the bile. The efflux of the cholesterol is controlled by adenosine triphosphate (ATP)-binding cassette sub-family G member 5 (ABCG5) and (ATP)-binding cassette sub-family G member 8 (ABCG8) encoded by *Abcg5* and *Abcg8* genes, respectively. They are mainly expressed in the liver and are up-regulated in response to high-cholesterol diet [51]. In this study, adding cholesterol to the diet significantly down-regulated hepatic *Abcg5* and *Abcg8* expressions. The suppression of these two genes by cholesterol would limit efflux of cholesterol to the bile duct, resulting in the elevation of serum cholesterol concentrations which was observed in the serum of the hamsters. Interestingly, feeding of *T. indica* fruit pulp to hypercholesterolaemic hamsters significantly up-regulated expression of *Abcg5*. This result suggests that *T. indica* fruit pulp stimulates *Abcg5* in response to the high-cholesterol diet intake to increase cholesterol secretion into the bile and inhibit cholesterol absorption, thus lowering serum cholesterol levels. Yu *et al*. [52] reported that over-expression of *Abcg5* and *Abcg8* in mice promoted cholesterol efflux and lowered plasma cholesterol concentrations. Transgenic mice expressing both human and mouse *Abcg5* and *Abcg8* genes had 50% reduction in cholesterol absorption and marked increased in biliary cholesterol and faecal neutral sterol secretion [53]. It has been suggested that *Abcg5* and *Abcg8* may work in concert as an apical sterol export pump to promote cholesterol and plant sterol efflux [51]. However, data from this study did not support this hypothesis as only the expression of *Abcg5* was up-regulated but there was no significant difference in *Abcg8* expression between hypercholesterolaemic hamsters and hypercholesterolaemic hamsters supplemented with the *T. indica* fruit pulp. Our findings support our previous *in vitro* study [10] where only *ABCG5* and not *ABCG8* was up-regulated in response to *T. indica* fruit pulp treatment of HepG2 cells.

Another gene that was studied was *Mtp* which encodes the heterodimeric microsomal triglyceride transfer protein (MTP). MTP is rate-limiting for assembly and secretion of apoB-containing lipoproteins such as very low density lipoprotein (VLDL-C) and chylomicrons [54], [55]. Diet enriched with cholesterol induced *Mtp* gene expression [56], [57]. In the present study, expression of *Mtp* gene was up-regulated in hypercholesterolaemic hamsters and this was accompanied by elevated triglyceride levels. It is possible that increased amounts of MTP are needed for the clearance of elevated triglyceride accumulated in the liver of hypercholesterolaemic hamsters. Bennett and Bruce [58] showed that hepatic *Mtp* gene expression and triglyceride levels increased in hamsters fed with cholesterol in a dose-dependent manner. Interestingly, the administration of *T. indica* fruit pulp to hypercholesterolaemic hamsters down regulated the expression of *Mtp* gene. This suggests that *T. indica* fruit pulp could provide lipid-lowering effect partially by decreasing hepatic *Mtp* gene expression, hence suppressing the assembly, accumulation and secretion of apo B-containing lipoproteins and triglyceride from liver to blood circulation. One of the active compounds in *T. indica*, naringenin, had been shown to reduce apoB accumulation in HepG2 cells via the inhibition of MTP activity and expression [59] and this may explain the reduction of triglyceride in the *T. indica* fruit pulp-treated hypercholesterolaemic hamsters.

In this study, we also tested the effects of *T. indica* fruit pulp on markers of oxidative stress and antioxidant activities as plants rich in antioxidants have the potential to protect against oxidative damage. CAT, SOD and GPx are antioxidant enzymes which play a significant role in the cellular defense against deleterious effects of free radicals. A decrease in the activities of these enzymes could increase the availability of O_2_^•^ and H_2_O_2_, which can lead to the production of OH^•^, subsequently initiating oxidative damage.

The reduced antioxidant activities as well as CAT, SOD and GPx activities in hypercholesterolaemic hamsters indicated possible oxidative stress conditions, leading to increased levels of O_2_^•^ and H_2_O_2_ CAT and SOD had been reported to be inactivated by ROS and lipid peroxides [60], [61]. Administration of *T. indica* fruit pulp to either control or hypercholesterolaemic hamsters increased antioxidant activities together with activities of CAT, SOD and GPx, although for GPx, this was only significant in the liver. Increased SOD implies increased capacity to detoxify O_2_^•^, leading to increased production of H_2_O_2_. Concurrently, increased CAT and GPx allowed for the effective removal of excess H_2_O_2_. Thus *T. indica* fruit pulp could prevent the cholesterol-induced oxidative stress by enhancing and restoring the antioxidant defense systems.

Malondialdehyde (MDA), the end product in the oxidative breakdown of unsaturated fatty acids, is a marker used frequently to evaluate lipid peroxidation in tissues. In the present study, the high-cholesterol diet induced lipid peroxidation, suggesting increased oxidative stress. This could be due to increased free radical formation accompanied by reduced antioxidant enzyme and antioxidant activities. Hypercholesterolaemia has been reported to elevate the levels of lipid peroxidation in hamsters [9]. Administration of *T. indica* fruit pulp along with the high-cholesterol diet inhibited lipid peroxidation, demonstrating the protective effects of *T. indica* fruit pulp against oxidative damage. The increased antioxidant activities and antioxidant enzymes could have also contributed towards the protective effects against lipid peroxidation. Phenolics such as catechin, procyanidins and naringenin that are present in *T. indica* fruit pulp have been reported to improve the antioxidative defense mechanism by increasing antioxidant activities, SOD and CAT and inhibiting lipid peroxidation in rats [62], [63].

Besides measuring the expression of genes in cholesterol metabolism, two genes (*Cyp1A1* and *Gstm1)* involved in xenobiotic metabolism were also evaluated. Their differential expression can provide beneficial information on the effect of the fruit pulp on antioxidant activities and oxidative stress. Xenobiotics and carcinogens are generally detoxified by phase I and phase II enzymes. Phase I detoxifying enzymes mainly composed of cytochrome P450 (CYP) supergene family of enzymes while phase II detoxifying enzymes comprised of glutathione S-transferase (GST) family. Variations in the expression of phase I and phase II detoxification enzymes may explain the carcinogenic effects leading to cancer. Hence, the influence of *T. indica* fruit pulp and cholesterol on the expression of genes related to detoxification was measured by qRT-PCR.

Cytochrome P450 Cyp1A1 is one of the members of the CYP family involved in the metabolism of drugs, environmental pollutants and carcinogens as well as a small number of endogenous substrates [64], [65]. In this study, hypercholesterolaemic hamsters showed down-regulation of hepatic *Cyp1A1* expression. However, *T. indica* fruit pulp administration to the hypercholesterolaemic hamsters reversed this condition. The induction of *Cyp1A1* might be essential to provide protection from the harmful effects of disruptors in the environment. Our study also showed that feeding of *T. indica* fruit pulp to hamsters induced *Cyp1A1* gene expression as compared to control hamsters. Quiñones and Lucas [66] reported that people carrying *CYP1A1* polymorphism could be more susceptible to lung cancer induced by environmental pollutants. This may indicate that *T. indica* fruit pulp has the potential to defend against carcinogenic risk via the induction of *Cyp1A1* gene expression. Although some studies have reported the carcinogenic effects of *Cyp1A1* [67], [68], more recent studies have implied potential detoxication as well as chemoprevention activities of *Cyp1A1* [69]. Several phytochemicals including the flavonoids quercetin was reported to induce the expression of *Cyp1A1* in MCF-7 breast cancer cells [70].

*Gstm1* encodes the glutathione S-transferase Mu 1, an enzyme involved in phase II detoxification of electrophilic compounds such as products of oxidative stress, environmental toxins and carcinogens and its impairment is associated with increased cancer risk [71]. The hepatic *Gstm1* gene expression level was significantly lowered in hamsters fed cholesterol diet compared with control. This signifies that cholesterol potentially inhibits the expression of *Gstm1,* thus enhancing the susceptibility of hypercholesterolaemic hamsters to environmental and carcinogenic challenges. Administration of *T. indica* fruit pulp to hypercholesterolaemic hamsters induced *Gstm1* expression to a level similar to control hamsters. In addition, administration of *T. indica* fruit pulp to control hamsters also increased the hepatic *Gstm1* gene expression significantly. These results suggest that *T. indica* fruit pulp could reverse the detrimental effects of high-cholesterol diet and contribute to the prevention of carcinogenesis by inducing the phase II detoxifying enzyme. Together, the regulation of both *Cyp1A1* and *Gstm1* by *T. indica* fruit pulp seems to be in a coordinated manner.

### Conclusion

In conclusion, this study shows that *T. indica* fruit pulp is a natural health food with hypocholesterolaemic and antioxidant properties. *T. indica* fruit pulp exerts its potential hypocholesterolaemic action by increasing hepatic gene expression of *Apo A1*, *Abcg5* and *LDL receptor* while suppressing *HMG-CoA reductase* and *Mtp* gene expressions. Hence, *T. indica* fruit pulp could potentially enhance cholesterol efflux, inhibit cholesterol biosynthesis, increase uptake and clearance of LDL-C from peripheral tissues and suppress triglyceride accumulation in the liver. On the other hand, supplementation of *T. indica* fruit pulp to hamsters did not lead to notable changes in lipid profiles compared to control hamsters. These observations imply that the fruit pulp is only beneficial under hypercholesterolaemic conditions. In addition, *T. Indica* fruit pulp can also prevent oxidative damage, particularly oxidation of LDL-C, which is one of the risk factors for atherosclerosis. These beneficial effects may be attributed to the phytochemical constituents, including the many phenolic and flavonoid compounds in the *T. indica* fruit pulp. *T. indica* fruit pulp may have clinical applications particularly in individuals with hypercholesterolaemia who may benefit from the cholesterol-lowering effect of this plant as well as its potential protection against oxidative damage. However, further work is needed to confirm the efficacy and proper dosage of the *T. indica* fruit pulp as a potential cholesterol-lowering agent including human trials to gain further insight into its therapeutic effect.
